# Supplementary material for: Identification and Functional Analysis of Drought-Responsive Long Noncoding RNAs in Maize Roots
Source: Int J Mol Sci. 2023 Oct 10;24(20):15039. doi: 10.3390/ijms242015039 (PMC10606207; doi:10.3390/ijms242015039)
Supplement: Supplementary file 1 [file ijms-24-15039-s001.zip › ijms-2550782-supplementary.pdf]

Supplementary Figures

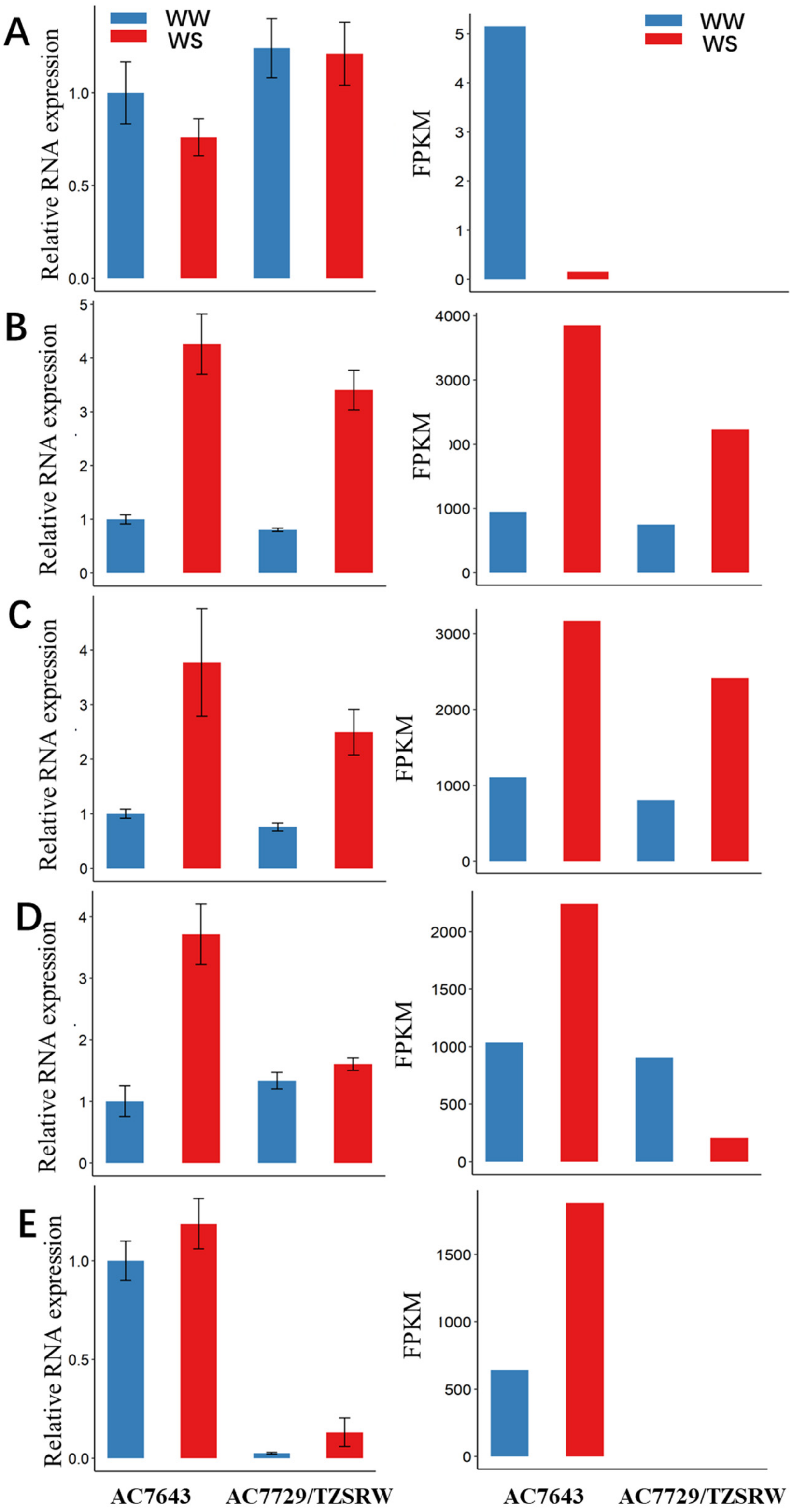

Supplementary Figure S1. The relative expression (Left) and FPKM (Right) of lncRNAs in different drought-tolerant materials (AC7643 and AC7729/TZSRW) under different water conditions (WW and WS). A：XLOC_007641，B：XLOC_032450，C：XLOC_017628， D：XLOC_027965，E：XLOC_028529.

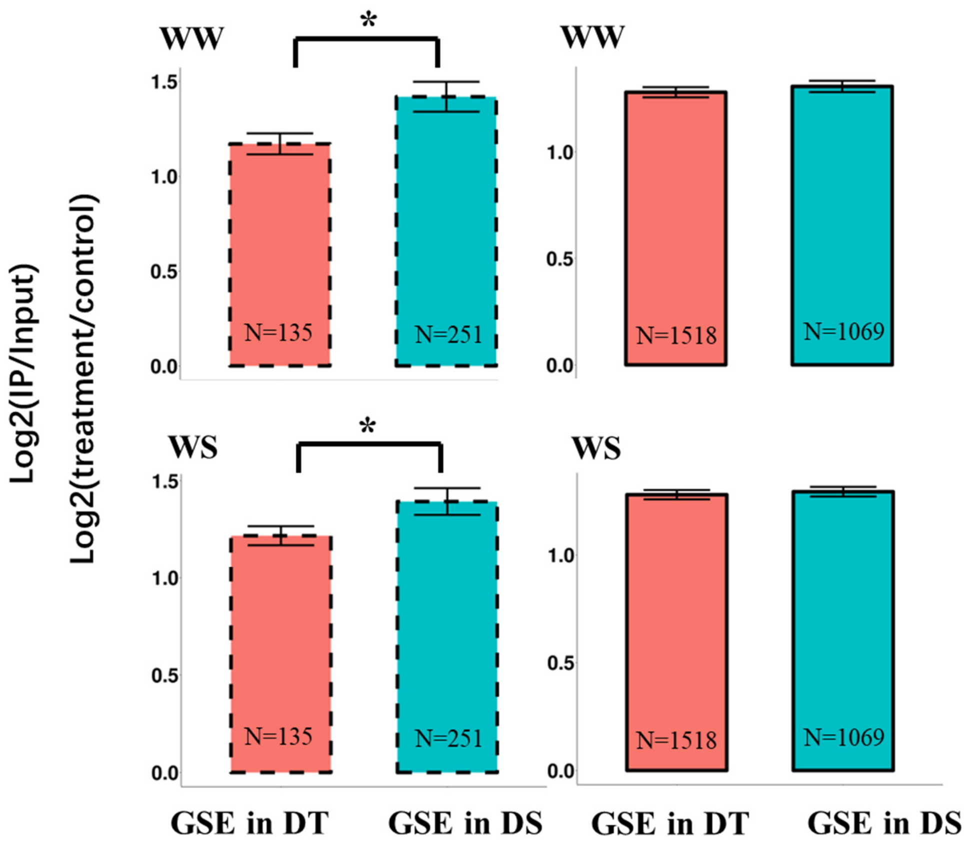

Supplementary Figure S2. The reads of H3K9AC modification at the genes specifically expressed in different dr ought resistant inbred lines under WW and WS. GSE in DT, genes specifically expressed in drought-tolerant inbred lines. GSE in DS, genes specifically expressed in drought-sensitive inbred lines. WW, well-watered; WS, water-stressed. *, $p < 0.05$.
